# Optical Designs with Curved Detectors for Fiber Bragg Grating Interrogation Monitors

**DOI:** 10.3390/s21010034

**Published:** 2020-12-23

**Authors:** Eduard Muslimov, Nadezhda Pavlycheva, Emmanuel Hugot, Simona Lombardo, Ilnur Nureev, Oleg Morozov

**Affiliations:** 1 Kazan National Research Technical University Named after A.N. Tupolev-KAI, 420111 Kazan, Russia; nkpavlycheva@kai.ru (N.P.); iinureev@kai.ru (I.N.); microoil@mail.ru (O.M.); 2 Aix Marseille University, CNRS, CNES, LAM, Groupe R&D Optique & Instrumentation, 13388 Marseille, France; emmanuel.hugot@lam.fr (E.H.); simona.lombardo@lam.fr (S.L.); 3 CURVE-S.A.S., 13388 Marseille, France

**Keywords:** fiber Bragg grating sensors, spectrograph, high angular dispersion, curved detectors, freeform optics

## Abstract

In this paper, we evaluate the application of curved detectors and freeform optics technologies for fiber Bragg gratings (FBGs) interrogation monitors design. It is shown that, in a high-dispersion spectrograph scheme, the camera part operates in special conditions, which result in a field curvature change. This field curvature can be compensated by the use of a curved detector. When used together with freeform optics, the curved detectors allow for reduction of the number of optical components to two or even one element by merging their functions. Three design examples for the range of 810–860 nm reaching the spectral resolution limit of 89–139 pm at NA=0.14 are presented to demonstrate the achieved performance and the technological trade-offs.

## 1. Introduction

The sensors based on fiber Bragg gratings (FBGs) have a number of advantages, which have been demonstrated for different applications [1]. They can be used for temperature and pressure measurements, while the sensors of both types can be combined in a single fiber. Their outstanding thermal range and electromagnetic stability makes them a primary choice for operations under harsh environments, like the aerospace [2] or the oil and gas [3] industries. The possibility to build a distributed sensing network of FBGs [4] encourages their application for structural health monitoring. The number of other application examples is impressively large, and it keeps growing.

The readout of FBG sensors requires measurement of their spectral response change with a very high accuracy. It can be done with interrogation monitors, which essentially represent high-resolution diffractive spectrographs with a photosensitive arrays. Such a device allows us to perform a simultaneous readout from tens of FBGs at high sensitivity without mechanical movements. But reaching a high spectral resolution in the dedicated waveband with limited size of the detector and instrument represents a separate engineering challenge.

In recent years, some technologies driving the field of imaging optics have experienced a fast growth. Namely, curved detectors technology has evolved from early concepts [5,6,7] to fully-functional instruments [8]. It has been proven that a complementary metal-oxide-semiconductor (CMOS) detector equivalent to a commercial flat one in terms of sensitivity and electronic performance can be curved to a desired shape with a high precision. Use of such a detector allows us to exclude the field curvature correction measures from the optical design, increase the resolution, and achieve a lower distortion and higher image illumination, as well as decrease the overall system dimension. In particular, it was demonstrated that the use of curved detectors makes it possible to significantly increase the performance of astronomical instrumentation and receive images of previously unresolvable objects [9,10]. Similar advantages apply to the technology of freeform optics [11], which has emerged during the last decade and allows to create optical systems notable for their outstanding resolution, high aperture, large field of view, and compactness.

The main goal of the present study was to explore a potential gain in performance and packaging achievable by applying freeform optics and curved detectors technologies in the optical designs of high-resolution spectrographs for FBG interrogation.

**Figure 1:**
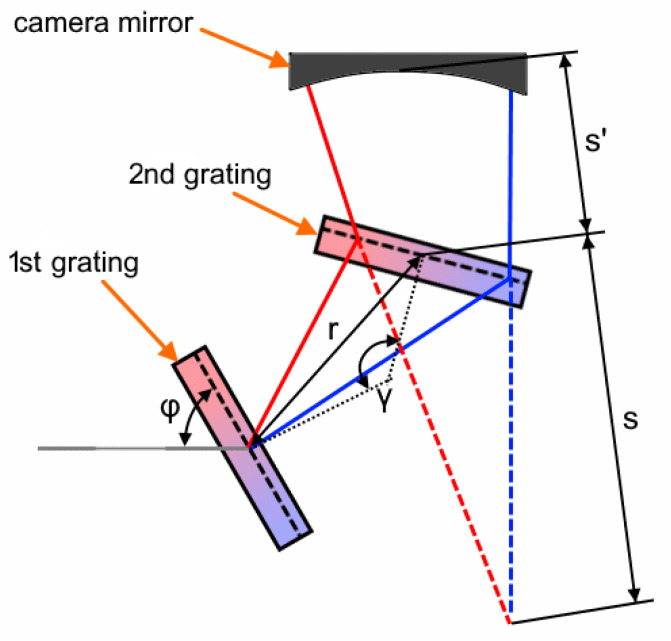
Chief ray tracing through a double dispersive unit. The *s + s’* distance corresponds to the equivalent pupil shift.

There are a few requirements intrinsic to the design of FBG interrogation monitors, which urged us to consider these technologies. First, there is a clear trend to decrease the number of optical components, thus reducing the size and increasing the environmental stability of the device. Use of freeform optics allows us to remove some optical components and merge a few functions in one element, while maintaining or increasing the optical performance. Second, reaching a high spectral resolution, together with a limited size, requires a high angular dispersion in a narrow spectral region. In some cases, such a high angular dispersion is achieved by using a complex dispersive unit representing a combination of gratings and/or prisms. Correction of the field curvature in such a design becomes an unusual task. The camera part is operating in a dispersed beam. To estimate the effect of dispersion on the field curvature, we consider an equivalent pupil offset in a dispersive unit with two transmission grating similar to that used in Reference [12]. It consists of two transmission gratings set up one after another to increase the angular dispersion in sequence (see Figure 1). In this figure, ϕ is the incidence angle on the first grating, γ is the angle between the gratings normals, *r* is the distance between the grating centers, and *s* and $s^{\prime }$ are the distances from the second grating center to the camera mirror and the equivalent pupil center, respectively. If we trace a chief ray at two different wavelengths through this unit, we can show that they have a virtual intersection point behind the second grating.

If the spectral image is focused by a single camera mirror, this setup is equivalent to a mirror working with a shifted pupil. Then, the field curvature $R_{m}$ can be approximately found from the following equation [13]:

(1) $$\frac{1}{R_{m}}=\frac{2}{R}-\frac{4\left[k\sigma^{2}+{\left(1-\sigma \right)}^{2}\right]}{R},$$

where *R* is the mirror vertex radius, *k* is the conic constant, and σ is

(2) $$\sigma =\frac{s+s^{\prime }}{R}.$$

**Figure 2:**
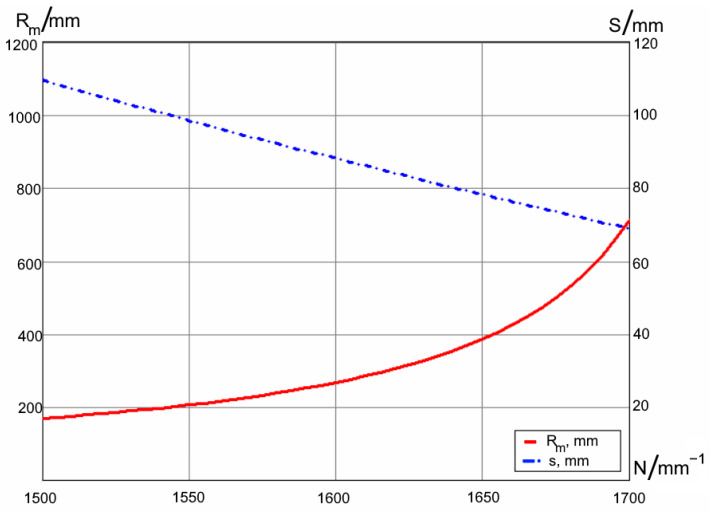
Equivalent pupil shift and the field curvature as a function of the grooves frequency.

We apply this procedure for the following typical set of parameters: wavelengths λ = 810–860 nm; angle between the gratings $\gamma =90^{\circ }$; incidence on the first grating $\phi =45^{\circ }$; distance between the gratings r=60 mm; distance to the mirror $s^{\prime }=50$ mm; mirror’s vertex radius R=−200 mm. The resultant equivalent pupil shift and the field curvature radius as functions of the gratings spatial frequency *N* are shown in Figure 2.

The plot shows that the ray’s intersection position and the curvature changes significantly with the dispersion. This effect must be taken into account in the spectrograph optical design.

Further, we consider a number of optical designs taking advantage of curved detectors and freeform optics. We gradually increase the level of design integration, merging a few functions in one optical element. In each case, we compare design versions with flat and curved detectors to show their impact. We use a design with a pair of transmission grating similar to that described in Reference [12] as the starting point. For all of the designs, we use the same set of basic parameters inspired by a commercial FBG interrogator [14] and accounting for parameters of an existing linear CMOS sensor [15] with 4096 pixels and a high sensitivity of 650 $\frac{V}{Lx\cdot s}$. The parameters are summarized in Table 1.

## 2. Optical Designs

**Figure 3:**
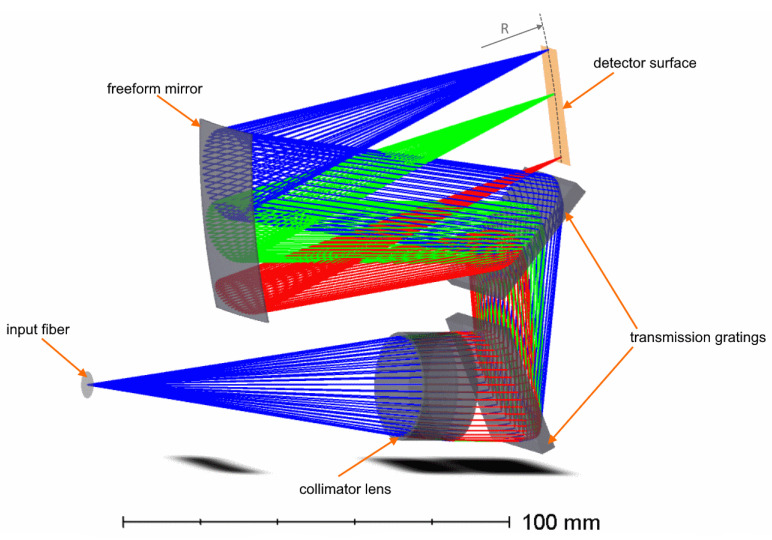
Optical design of the high-dispersion spectrograph with a pair of transmission gratings (Design A).

The first optical design under consideration consists of a doublet lens collimator, two transmission gratings, and a freeform mirror acting as a camera objective. The gratings have straight equally spaced grooves and are imposed on flat surfaces. For simplicity, we assume that they have the same grooves frequency, equal to 1639 mm${}^{-1}$. The freeform mirror shape is described by Zernike polynomials. The general view of the optical design is shown in Figure 3. Hereafter, this design is denoted as “Design A”. For this case, Equations (1)–(2) can be applied directly. We applied them to obtain the first estimation of the field curvature and then repeated optimization with curved and flat detectors. It appears that the curvature can be efficiently compensated by the freeform, since the image surface radius in the resultant design is –5134 mm.

**Figure 4:**
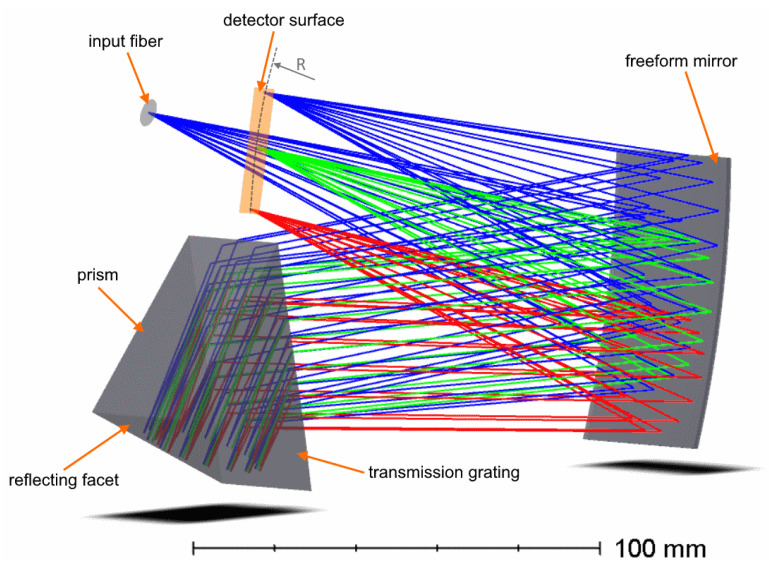
Optical design of the high-dispersion spectrograph with a double-pass immersed grating (Design B).

In the second design (“Design B”), some functions are performed by the same optical element. Thus, the dispersive unit has only one grating working in a double-pass mode to increase the dispersion. In order to create this double-pass geometry a transmission grating with spatial frequency of 1900 mm${}^{-1}$ is imposed on the first surface of the prism. The prism is made of Schott N-LASF41 (glass catalogue of Schott AG, Mainz, Germany) glass and has an apex angle of $59.3^{\circ }$. The prism rear surface has a reflective coating. This solution is to some extent similar to immersed gratings proposed in Reference [16,17]. The freeform mirror in Design B serves as both the collimator and camera. To avoid geometrical conflicts between the beams in forward and backwards propagation, a sagittal shift of the source is used. The fiber input has the sagittal coordinate x=−5 mm, so the image coordinate is $x^{\prime }=5$ mm. The layout of Design B is shown in Figure 4. To find the image curvature, we found the prism geometrical development, found the initial value of the field curvature, and performed numerical optimization for designs with flat and curved detectors, as it was done for the “Design A”. The optimal image radius found after optimization is 186.2 mm.

**Figure 5:**
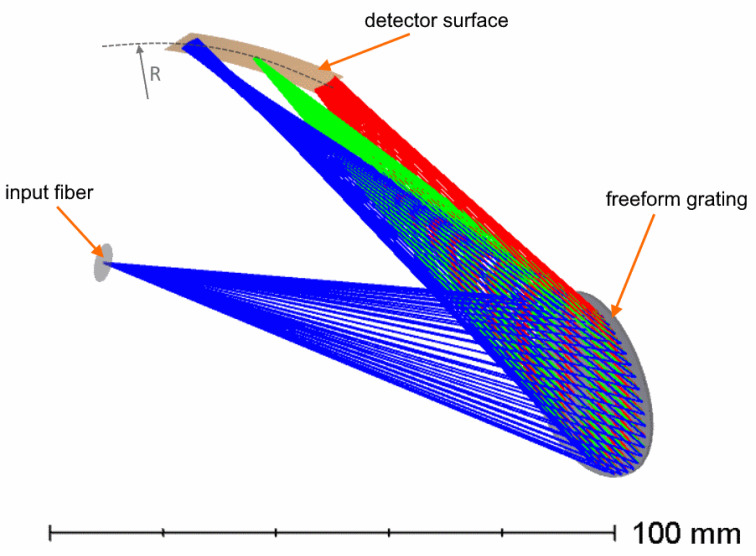
Optical design of the high-dispersion spectrograph with a freeform grating (Design C).

Finally, we consider combining all the functions in one optical element: a grating with non-equally spaced grooves imposed on a concave XY-polynomial freeform surface working as the collimator, dispersive unit, and camera at the same time. It becomes practically impossible to use a double-dispersion geometry, so the grating has spatial frequency of 1623 ${\mathrm{mm}}^{-1}$ in its vertex and works with high dispersion angles. This design (“Design C”) is presented in Figure 5. This design follows the model of spectrographs on Rowland circle [18], although the number of correction parameters is much higher. In this case, the pupil coincides with the grating and the spectral image lies on a cylindrical surface with radius of R/2. The radius found after optimization is 107.9 mm.

## 3. Imaging Performance Assessment

**Figure 6:**
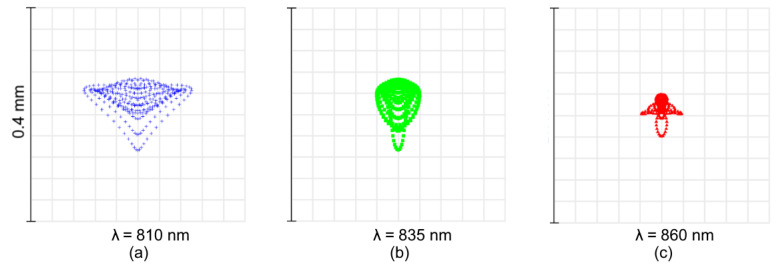
Spot diagrams of the high-dispersion spectrograph Design A.

The imaging performance of each design is estimated through the spot diagrams and instrument functions at three reference wavelengths, corresponding to the center and edges of the working range. The spot diagrams for Design A are shown in Figure 6.

The diagrams show that the aberrations are corrected in both tangential and sagittal plane. The image quality is high over the entire range (Figure 6a–c), although the longwave edge (Figure 6c) is slightly over-corrected. The root mean square (RMS) spot radius varies from 25 to 63 μm, while the maximum radii are 38–111 μm.

**Figure 7:**
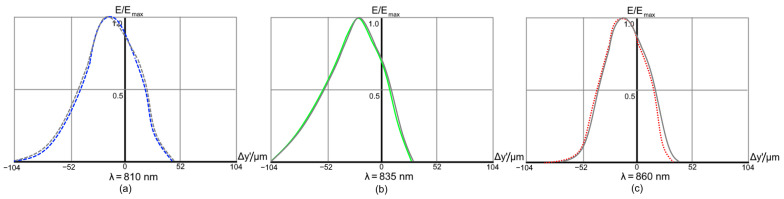
Instrument functions of the high-dispersion spectrograph Design A for a 50 μm input fiber. The grey lines correspond to the design with flat detector.

The instrument function (IF) is computed for an input fiber width of 50 μm. The IF graphs, i.e., the relative illumination in the monochromatic image of fiber end at each of the three reference wavelengths, are shown in Figure 7a–c. Here, the results obtained with a flat detector are shown in grey for comparison. It is clear that, in this particular case, the freeform camera provides a sufficient aberration correction, and the curved sensor contribution is as low as 2.6%.

For the given values (see Table 1), the average reciprocal linear dispersion is 1.74 nm/mm. This value, together with the pixel size, defines the device spectral sensitivity. For instance, a pixel size of 7 μm is equivalent to the spectral shift of 12 pm or 0.012 nm. The product of the IF full-width-at-the-half-maximum (FWHM) by the reciprocal linear dispersion represents the spectral resolution limit. It affects both the sensitivity to the spectral shift and also the number of FBG sensors, which can be measured simultaneously. The spectral resolution limit values for all the designs are summarized in Table 2.

**Figure 8:**
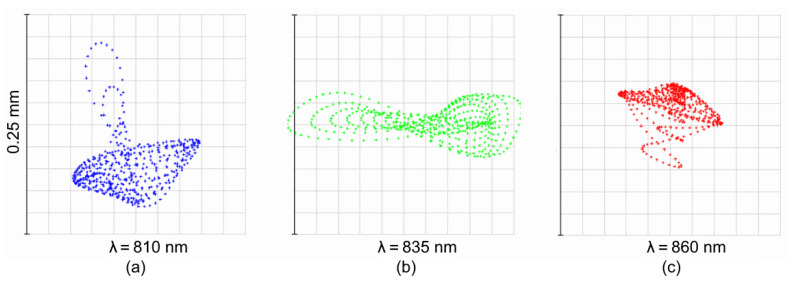
Spot diagrams of the high-dispersion spectrograph Design B.

Similarly, the spot diagrams for Design B are given in Figure 8. The plots show that the aberration correction is good and uniform, though the spots are more affected by high-order aberrations. The RMS radii are 43–84 μm, and the maximum radii are 71–144 μm. However, the spot at central wavelenght is elongated in the sagittal direction (Figure 8b) and the spots blurring in tangential direction at the edges (Figure 8a,c) is negligible. So the spectral resolution remains high.

**Figure 9:**
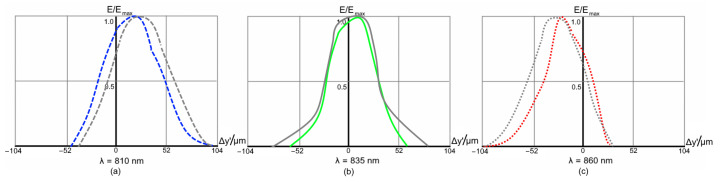
Instrument functions of the high-dispersion spectrograph Design B for a 50 μm input fiber. The grey lines correspond to the design with flat detector.

The Design B IF graphs are shown in Figure 9. The resolution is compatible to that achieved in Figure 7, but the effect of the curved detector is more significant. The use of a curved image plane allows to gain up to 23% in terms of resolution (see Figure 9c).

**Figure 10:**
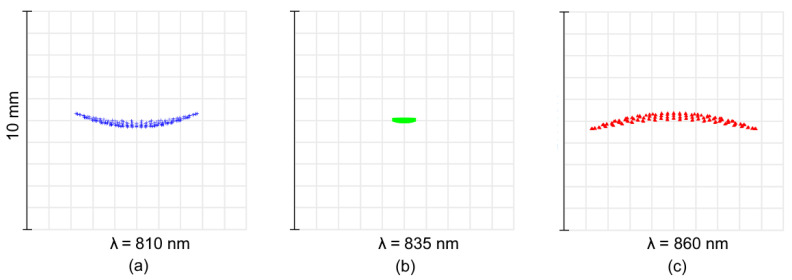
Spot diagrams of the high-dispersion spectrograph Design C.

Finally, the spot diagrams of Design C are presented in Figure 10. It is clear that the design is driven by a high astigmatism, intrinsic for the parent Rowland circle-based solution. It is corrected only for the central wavelength (Figure 10b). The astigmatic elongation of the spot image at the edges of spectral range reaches ±3.8 mm (Figure 10c). However, in the spot dimension, the tangential section remains relatively small and varies from ±43 to ±290μm.

**Figure 11:**
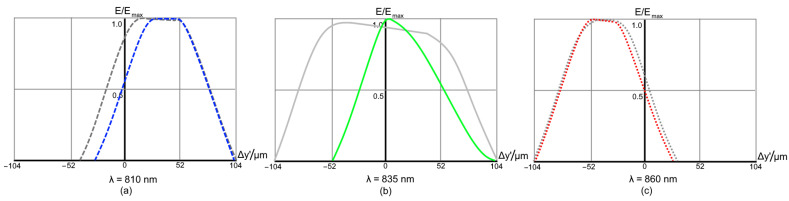
Instrument functions of the high-dispersion spectrograph Design C for a 50 μm input fiber. The grey lines correspond to the design with flat detector.

The corresponding IFs are shown in Figure 11. In this case, the gain in resolution obtained by using a curved detector is as high as 92.3% (see Figure 11b).

Table 2 summarizes all of the computed values of the spectral resolution limit. For each design and each reference wavelength, the values with curved/flat detectors are given.

## 4. Components Analysis

In the present section, we investigate the parameters of the freeform mirrors and curved detectors found during the optimization in more detail. The main purpose of this analysis is to assess their complexity and technological feasibility.

### 4.1. Freeform Elements

**Figure 12:**
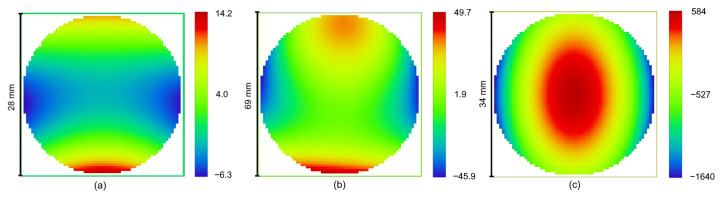
Deviation from the best fit sphere (in microns) of the freeform mirrors surfaces.

In designs A and B, the freeform surface shape was described by the standard Zernike polynomials:

(3) $$z=\frac{cr^{2}}{1+\sqrt{1-\left(1+k\right)c^{2}r^{2}}}+\sum_{i=1}^{n}A_{i}Z_{i}\left(\rho ,\phi \right),$$

where *c* is the vertex curvature, *r* is the radial coordinate on the surface, *k* is the conic constant, $Z_{i}$ are the Zernike polynomials, and $A_{i}$ are the corresponding coefficients. In both cases, the number of polynomials was limited by the 5th order. Since the design A is symmetric with respect to the YZ plane, only 9 Zernike symmetrical modes were used. Design B brakes the plane symmetry to separate the incoming and outcoming beams, so additional modes, namely $Z_{5}$ and $Z_{8}$, were introduced. The residual surface sags after subtracting the best fit sphere for the freeform surfaces in Designs A and B are shown in Figure 12a,b, respectively. The corresponding numerical estimates are given in Table 3.

In design C, the surface of the freeform grating is described by the following equation:

(4) $$z=\frac{c(a^{2}x^{2}+b^{2}y^{2})}{1+\sqrt{1-a^{2}x^{2}-b^{2}y^{2}}}+\sum_{i=1}^{n}A_{i}E_{i}\left(x,y\right).$$

Here, a,b, and *c* are semi-axes of the ellipsoid, $E_{i}$ are non-normalized XY-polynomials, and $A_{i}$ are the corresponding coefficients. Although use of this equation may decrease the optimization efficiency, it allows to apply standard modeling features. Eight XY polynomials, including all the YZ-symmetric modes up to the 4th order, were used. The freeform shape is also presented in Figure 12c and Table 3. The surface shape is driven by the large astigmatism of Rowland-type mounting. It causes a high difference between the curvatures in tangential and sagittal directions, so it becomes impossible to find the BFS, and, as a result, a plane is used as the reference shape.

The grooves pattern of the grating in Design C has a varying period, which follows the law:

(5) $$\frac{1}{N}=\frac{1}{N_{0}}+\alpha y+\beta y^{2}+\Gamma y^{3}+\Delta y^{4}+\epsilon y^{5},$$

where $N_{0}$ is the grooves frequency in the vertex in $\mu m^{-1}$, α−ϵ are the non-uniformity coefficients and *y* is the coordinate of point on the grating surface in mm. Such a pattern can be formed by holographic recording or etched through a mask. The values found by optimization are: $\alpha =6.518\times 10^{-4}\;\frac{\mu m}{\mathrm{mm}},\beta =1.718\times 10^{-5}\;\frac{\mu m}{{\mathrm{mm}}^{2}},\gamma =5.184\times 10^{-7}\;\frac{\mu m}{{\mathrm{mm}}^{3}},\delta =3.314\times 10^{-9}\;\frac{\mu m}{{\mathrm{mm}}^{4}},\epsilon =2.990\times 10^{-11}\;\frac{\mu m}{{\mathrm{mm}}^{5}}$. Applying these coefficients in Equation (5) results in varying of the grating period by 0.042 μm, or 6.4%, which is technologically achievable [19].

The data indicate that the freeforms designs A and B are both driven by the primary coma and astigmatism and have slowly varying sag with a moderate peak deviation. Both of them should be feasible with the current level of the freeform polishing technology [20]. One may also note that the freeform in the design B is slightly asymmetrical to compensate the beam deviation from the YZ plane. The freeform shape obtained in the design C appears to be more challenging. However, the surface is relatively smooth; the higher XY orders contribute to the total sag only by 23.7 μm or 1.1%.

### 4.2. Curved Detectors

**Figure 13:**
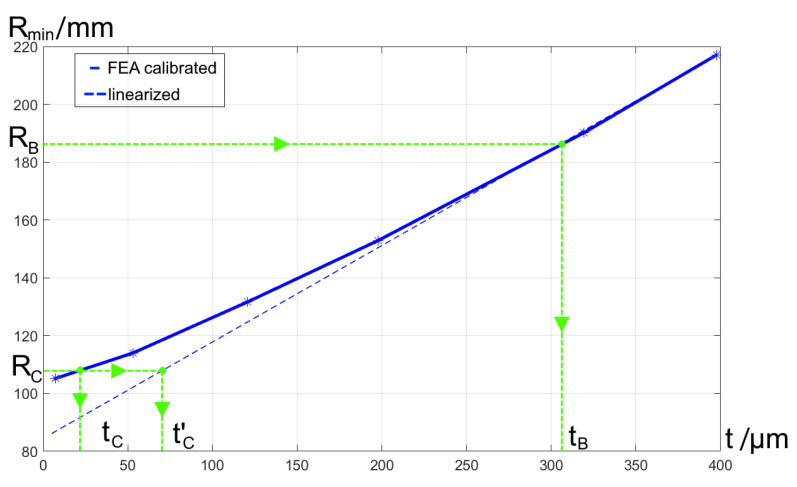
Dependence of the achievable detector radius of curvature on the dye thickness.

The feasibility of the found curved detectors shapes can be estimated through the required thickness of the silicon dye. The curving process was modeled in Reference [21], both analytically and numerically by finite-element analysis. As a result, a set of calibration curves was produced. They define the minimum radius of curvature, which can be achieved for a sample of given length and thickness without risk of breakage. The calibration curves for a 28-mm length sample are shown in Figure 13. The optimal detector radius in design A is extremely large, so it can be considered as flat. Applying the calibration curve for the detector radius in design B, which equals to $R_{B}=186.2$ mm, we find the required thickness of $t_{b}=$ 306.4 μm. This value is technologically achievable. Repeating the same procedure for the design C radius of $R_{C}=107.9$ mm, we obtain the thickness equal to 21.9 μm. This value is practically unreachable with the current back-thinning process. This means that the existing curving process, which was developed to produce spherically shaped image detectors, cannot be applied in this case directly. However, typical spectrographs work with linear detector arrays. This implies that the size of detector in sagittal direction can be smaller than that in tangential direction by two orders of magnitude. In this case, one can neglect the curvature in the sagittal plane and consider curving the detector into a cylindrical shape instead of a spherical one. A cylindrical curving generates less mechanical stress, and a simplified linear model can be used to compute the required thickness. Referring to the linearized model from the same source [21] (see the dashed line in Figure 13), we find the dye thickness of $t_{C}^{\prime }=$ 70.6 μm. This value is close enough to the thicknesses obtained with the existing technology. This implies that the computed detector shape is technically feasible but requires some revision of the curving process to generate a cylindrical shape instead of a spherical one.

## 5. Results and Discussion

In the previous sections, it was shown that field curvature aberration is intrinsic for high dispersion spectrographs and that they can be successfully eliminated by the use of curved detectors. In addition, it has been demonstrated that application of the freeform optics technologies allows us to create new optical designs with merged functionality of the components. The imaging performance analysis indicates that the new designs provide high image quality, while decreasing the number of components and the overall dimensions. The research stage presented in this paper is focused on comparison of the optical design concepts and estimation of the performance gain reachable with the freeforms and curved detectors. But, it is also important to assess the performance difference for a certain target application, i.e., for FBG response sensing. It would be practically difficult and even excessive to make an experimental proof for each of the proposed designs, so we have to rely on simulations.

**Figure 14:**
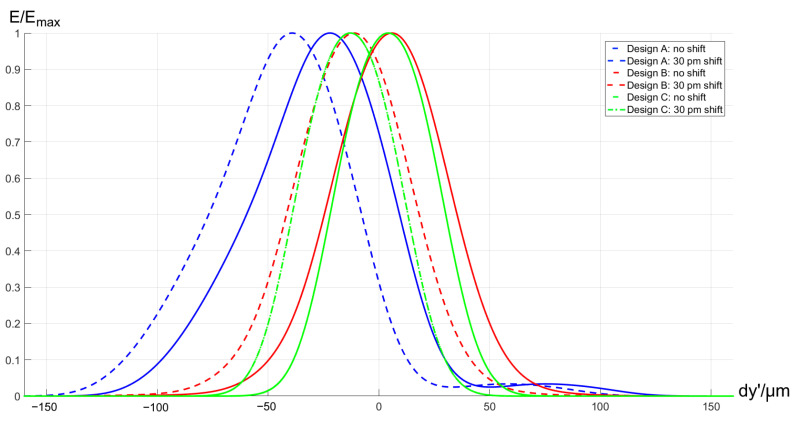
Simulation of fiber Bragg grating (FBG) profiles obtained with different designs.

We consider an FBG, with a Gaussian profile of 0.05 nm FWHM centered around 835 nm [22]. We take the narrowest profile for simulations to make the difference more visible. It is recomputed to an ideal image profile with the scale of spectrograph linear dispersion equal to 1.74 nm/mm. Then, it is convolved with the 50 μm-width rectangular function representing the input fiber acting as a spatial filter. Finally, the result is convolved with the line spread functions (LSF) computed for each design. The computation is repeated for a centered FBG signal and a signal spectrally shifted by 30 pm. The results of the image simulations are shown in Figure 14.

The plot shows that the curve displacement corresponding to the signal spectral shift is identical for all the designs. The output curve for Design A has a notable asymmetrical broadening with FWHM of 69.1μm, 9.9% of FWHM asymmetry, and lateral offset of 21.7μm due to the coma-type aberration. In addition, it has a 3.3% pedestal-type artifact at the bottom part. All of these imperfections are signatures of the optical design. The offset effect may require an additional calibration, while the curve asymmetry and broadening will decrease the precision of the line position measurement, and the artifact will decrease the maximum number of sensors, which can be readout at the same time.

The simulated output for Design B is almost free of the aberrations signatures. It has a minor offset of 1.0μm and a residual symmetrical broadening with 60.04μm FWHM and 1.03% asymmetry.

The signal for Design C is even closer to the initial convolution of Gaussian profile and rectangular function. The offset is 0.02μm, the broadened FWHM is 51.9μm, and the asymmetry is 0.02%, which is negligible.

Thus, all of the developed designs can be applied for the FBG sensing tasks. Design A is notable for using simpler optics and low field curvature. However, it uses more optical components, has larger dimensions and demonstrates decentering and broadening in the output due to the residual aberrations. Design B uses less components and has a reduced volume together with a better imaging performance in an FBG sensing application. This solution may be more sensitive to manufacturing errors because of its double-pass geometry. In addition, such an off-plane design is challenging for development of the mechanical design and performing the alignment. Finally, Design C shows a superior imaging performance and consists of only two components. This result is achieved by the use of technologically challenging freeform grating and cylindrical curved detector, as well as by allowing a relatively high astigmatic elongation of the image.

## 6. Conclusions

In the present paper, we considered the prospects of application of freeform optics and curved detectors technologies in high-resolution spectrographs to be used for FBG sensors readout. We considered a high-dispersion spectrograph design with two transmission gratings for the range of 810–860 nm. The optimization and modeling results have shown that using of a freeform camera mirror in this design allows to correct the aberrations and reach a spectral resolution limit up to 89 pm.

Simultaneous application of the freeform optics and curved detectors technologies makes it possible to merge the optical components functions and create a design of two components: a freeform focusing mirror and a double-passed immersed grating. To the best of our knowledge, the proposed design is new for this type of application. This solution has approximately the same optical performance, and both the freeform and curved detector are feasible with the current technological level.

Finally, it was shown that using a freeform grating with a complex grooves pattern and a curved detector allows us to revise a classical design with a concave grating in a Rowland-circle mounting. This design consists of only one optical element and allows us to reach spectral a resolution limit of 139 pm. The grating, in this case, has an astigmatism-driven freeform shape with low contribution of the high orders, while the detector has a high curvature, which can be achieved for a linear detector curved into a cylindrical shape.

It was demonstrated by means of simulation that all of the designs exhibit a good performance in an FBG sensing application. Each of them has some intrinsic advantages and drawbacks in terms of image quality, size, and technological complexity. Depending of the practical priorities, one of these designs can be chosen for further investigations, including practical implementation and experimental studies.

## Figures and Tables

**Table 1:** Initial values for the spectrographs designs.

Parameter	Value
Working spectral range/nm	810–860
Input NA	0.14
Detector length/mm	28.7
Pixel size/μm	7 × 200
Collimator and camera focal lengths/mm	100

**Table 2:** Spectral resolution limit with curved/flat detector (in pm).

	Design A	Design B	Design C
810 nm	103/106	109/109	89/89
835 nm	120/120	92/98	89/109
860 nm	145/170	172/273	139/148

**Table 3:** Freefrom mirros asphericity data.

	Design A	Design B	Design B
Type	Zernike sag	Zernike sag	Ellipsoid + XY pol.
Diameter/mm	28.3	68.8	34.0
BFS radius/mm	195.9	282.5	flat
Max. BFS deviation/μm	14.2	29.7	543.6
RMS BFS deviation/μm	3.6	9.8	1636.7
